# Comparison of CT findings of coronavirus disease 2019 (COVID-19) pneumonia caused by different major variants

**DOI:** 10.1007/s11604-022-01301-1

**Published:** 2022-06-28

**Authors:** Shohei Inui, Akira Fujikawa, Wataru Gonoi, Shuichi Kawano, Keita Sakurai, Yuto Uchida, Masanori Ishida, Osamu Abe

**Affiliations:** 1 Department of Radiology, Graduate School of Medicine, The University of Tokyo, 7-3-1, Hongo, Bunkyo-ku, Tokyo, 113-8655 Japan; 2 Department of Radiology, Japan Self-Defense Forces Central Hospital, 1-2-24, Ikejiri, Setagaya-ku, Tokyo, 154-0001 Japan; 3 Department of Respiratory Medicine, Japan Self-Defense Forces Central Hospital, 1-2-24, Ikejiri, Setagaya-ku, Tokyo, 154-0001 Japan; 4 Department of Radiology, National Center for Geriatrics and Gerontology, 7-430 Morioka-cho, Obu, Aichi, 474-8511 Japan; 5 Department of Neurology, Graduate School of Medicine, Nagoya City University, 1 Kawasumi, Mizuho-cho, Mizuho-ku, Nagoya, Aichi, 467-8601 Japan

**Keywords:** SARS-CoV-2, 2019 novel coronavirus, COVID-19, CT, Alpha variant, Delta variant

## Abstract

**Purpose:**

To explore the CT findings and pneumonnia progression pattern of the Alpha and Delta variants of SARS-CoV-2 by comparing them with the pre-existing wild type.

**Method:**

In this retrospective comparative study, a total of 392 patients with COVID-19 were included: 118 patients with wild type (70 men, 56.8 ± 20.7 years), 137 with Alpha variant (93 men, 49.4 ± 17.0 years), and 137 with Delta variant (94 men, 45.4 ± 12.4). Chest CT evaluation included opacities and repairing changes as well as lesion distribution and laterality. Chest CT severity score was also calculated. These parameters were statistically compared across the variants.

**Results:**

Ground glass opacity (GGO) with consolidation and repairing changes were more frequent in the order of Delta variant, Alpha variant, and wild type throughout the disease course. Delta variant showed GGO with consolidation more conspicuously than did the other two on days 1–4 (vs. wild type, Bonferroni corrected *p* = 0.01; vs. Alpha variant, Bonferroni corrected *p* = 0.003) and days 5–8 (vs. wild type, Bonferroni corrected *p* < 0.001; vs. Alpha variant, Bonferroni corrected-*p* = 0.003). Total lung CT severity scores of Delta variant were higher than those of wild type on days 1–4 and 5–8 (Bonferroni corrected *p* = 0.01 and Bonferroni corrected *p* = 0.005, respectively) and that of Alpha variant on days 1–4 (Bonferroni corrected *p* = 0.002). There was no difference in the CT findings between wild type and Alpha variant.

**Conclusions:**

Pneumonia progression of Delta variant may be more rapid and severe in the early stage than in the other two.

## Introduction

New genetic lineages of SARS-CoV-2 have been emerging and circulating around the world, causing a persistent COVID-19 pandemic. Since the first detection of a SARS-CoV-2 variant case in Japan on December 25, 2020, the spread of different COVID-19 variants has caused a serious public health concern throughout the country [1]. Especially in 2021, the country suffered the fourth wave of the COVID-19 epidemic from April to May 2021 and the fifth wave from July to September 2021 [2]. During these periods, the pre-existing virus was rapidly replaced by the SARS-CoV-2 variants known as B.1.1.7 (Alpha variant) and B.1.351 (Delta variant), respectively [2].

These variants contain mutations in their spike proteins, resulting in substantial changes in their epidemiological and clinical profiles [3]. The risk of transmission was 45–71% higher for Alpha variant and < 50% higher for Delta variant than the pre-existing wild type [4–6] The risks of hospitalization, ICU admission, and mortality of Alpha variant were 1.53, 1.74, and 1.37 times of those of the wild type, respectively, and 2.08, 3.35, and 2.33 times, respectively, for Delta variant [7].

To date, two studies have investigated the radiological differences between the variant types of COVID-19. One from the UK detected no difference in the CT severity score between patients infected with Alpha variant and wild type [8] The other one from China revealed the chest CT findings of children infected with Delta variant to be milder and to improve more quickly as compared to the wild type [9]. However, the CT findings of variant types of COVID-19 have yet to be sufficiently investigated especially in adult Asian populations. This prompted us to undertake the present study to systematically investigate the CT findings of Alpha and Delta variants by comparing them with those of the wild type and each other.

## Materials and methods

This study was conducted with the approval of our institutional ethics review board (03-015). Written informed consent was waived due to the retrospective nature of the study. The privacy of all patients was protected.

### Study population

Patient backgrounds were standardized by applying the following inclusion criteria: (1) admitted to a single institution in Tokyo, Japan after being confirmed with COVID-19 with either real-time reverse transcription-polymerase chain reaction (RT-PCR) or rapid antigen test, (2) underwent at least one chest CT during hospital stay, (3) with clinical severity of mild or moderate based on the guidance statement issued by the Ministry of Health, Labour and Welfare [10]. Those categorized as severe case requiring ICU admission or mechanical ventilation were excluded. According to the weekly surveillance report on SARS-CoV-2 variant issued by a local municipal bureau in Tokyo (the Bureau of Social Welfare and Public Health), the majority of community-acquired cases were Alpha variant from April 19 to July 18 in 2021 and Delta variant from July19 to September 26 in 2021 [2]. Therefore, those who were being confirmed during the above-described periods were deemed to be infected with the variants dominating in the respective periods (hereafter referred to as Alpha variant and Delta variant, respectively). For wild type subgroup (hereafter referred to as wild type), conveniently sampled patients who were diagnosed with COVID-19 from September 1 to December 24 in 2020 before the first case of variant type of COVID-19 was confirmed in Japan were recruited. The study population comprised 118 patients (70 men; mean age, 56.8 years ± 20.7) with 128 CT scans for wild type, 137 patients (93 men; mean age, 49.4 years ± 17.0) with 146 CT scans for Alpha variant, and 137 patients (94 men; mean age, 45.4 years ± 12.4) with 140 CT scans for Delta variant.

### Clinical data

Medical records were reviewed for the clinical and imaging findings of the subject patients. The following data were extracted from the medical records: age, gender, duration from onset to CT, symptoms and signs, underlying comorbidities, and smoking history. Disease severities were defined according to the guidance statement issued by the Ministry of Health, Labour and Welfare [10]. Disease severity was defined as mild if one had SpO2 ≥ 96% and without respiratory symptoms other than cough and not requiring oxygen administration; and moderate if one had SpO2 < 96% and dyspnea on breath (DOB) and pneumonia and requiring oxygen administration.

### Chest CT acquisition

Enhanced or non-enhanced CT was performed using a 256-row multi-detector CT unit (Revolution CT; GE Healthcare, Milwaukee, WI, USA) with the following parameters: tube voltage, 100 kVp; collimation, 0.625 mm, helical pitch, 1.375, field of view, 36 cm; matrix size, 512 × 512 with optimized effective current under automatic exposure control (GSI Assist; GE Healthcare) based on the x-ray attenuation on anterior–posterior and lateral scout images. CT images were acquired during a single inspiratory breath-hold. A 2.0-mm gapless section was reconstructed for chest CT images before being reviewed on the picture archiving and communication system (PACS) monitor.

### CT image interpretation

Image analysis was performed on a PACS monitor independently by two board-certified radiologists (A.F. and S.I., with 33 and 7 years of experience, respectively), who were blinded to the clinical data including patient management and COVID-19 subtype. Interobserver disagreements were resolved by consensus during a joint reading to determine the results. Parameters evaluated included: primary change, i.e., either one of pure ground-glass opacity (GGO), GGO with reticular opacity (crazy-paving pattern), or GGO with consolidation, or absence of the above lesions; repairing change, i.e., fibrotic strips, bronchial distortion, or subpleural line. Axial zonal distributions of the lesions were classified as peripheral predominantly (i.e., involving mainly the peripheral one-half of the lung), central predominantly (i.e., involving mainly the central one-half of the lung), or both. The numbers of affected lobes and laterality of the lesions were also evaluated. The extension of the lung opacities was evaluated using a semiquantitative chest CT severity score, with which lung lesions were estimated as the percentage per lobe [11]. For each lung, the extent of anatomical involvement was visually scored on a scale of 0–5 as follows: score 1, 1–5% involvement; score2, 6–25% involvement; score 3, 26–50% involvement; score 4, 51–75% involvement; and score 5, 76–100% involvement. Total lung scores were calculated as the sum of the individual lobe scores. The presence or absence of reversed halo sign, centrilobular nodule, tree-in-bud, pleural effusion, thoracic lymphadenopathy (as defined by lymph node size of ≥ 10 mm in short-axis dimension), and fatty liver was also assessed. CT findings were compared after being stratified by chronological disease stages (days 1–4, days 5–8, days 9–12, and days 13 or more).

### Statistical analysis

Quantitative variables were expressed as mean ± standard deviation or median (interquartile range) based on the normality of data as defined by the Shapiro–Wilk test. Categorical variables were presented as the percentage of the total. The comparisons of quantitative variables were performed using a one-way repeated measures analysis of variance (ANOVA), Kruskal–Wallis test, Student's *t*-test or Mann–Whitney *U* test based on the normality of data and categorical data using the Pearson *χ*^2^ test or Fisher’s exact test. Post hoc family-wise error correction for multiple comparisons was adjusted using the Bonferroni method. Statistical analysis was done using the Python environment (version 3.7.3) by using SciPy (version 1.2.1) [12]. All *P* values correspond to two-sided tests and the statistical significance level was set at Bonferroni corrected *P* < 0.05.

## Results

### Clinical findings

Demographics and clinical characteristics of the study population were summarized in Table 1. Patient age was significantly different between subgroups with more aged patients in wild type (all, *p* < 0.001; wild type vs. Alpha variant, Bonferroni corrected *p* = 0.01; wild type vs. Delta variant, Bonferroni corrected *p* < 0.001; Alpha variant vs. Delta variant, Bonferroni corrected *p* = 0.3). Clinical severity was significantly different between subgroups with more moderate patients in Delta variant than the other two (all, *p* = 0.001; wild type vs. Alpha variant, Bonferroni corrected *p* = 1.0; wild type vs. Delta variant, Bonferroni corrected *p* = 0.003; Alpha variant vs. Delta variant, Bonferroni corrected *p* = 0.03). Cardiovascular diseases was the only comorbidities that differed significantly being more frequent in wild type than the other two (all, *p* < 0.001; wild type vs. Alpha variant, Bonferroni corrected *p* = 0.03; wild type vs. Delta variant, Bonferroni corrected *p* < 0.001; Alpha variant vs. Delta variant, Bonferroni corrected *p* = 0.3). There was no statistically significant difference in gender, onset to CT, symptoms, comorbidities other than cardiovascular diseases, or smoking status.

**Table 1:** Demographic data of this study cohort

	Wild type (*n* = 118)	Alpha variant (*n* = 137)	Delta variant (*n* = 137)	*p *value
Age	56.8 ± 20.7^d^	49.4 ± 17^d^	45.4 ± 12.4^d^	< 0.001^a^
Gender				
Male	70 (59%)	93 (68%)	94 (69%)	0.2^c^
Female	48 (41%)	44 (32%)	43 (31%)	–
Onset to CT (days)	7 [3–7]	7 [4–8]	8 [4–9]	0.08^b^
Clinical severity				
Mild	88 (75%)^e^	95 (69%)^e^	74 (54%)^e^	0.001^c^
Moderate	30 (25%)	42 (31%)	64 (46%)	–
Symptoms				
Fever (> 37.5 ℃)	101 (86%)	99 (72%)	92 (67%)	0.3^c^
Cough	70 (59%)	78 (57%)	95 (69%)	0.08^c^
DOB	43 (36%)	37 (27%)	50 (37%)	0.2^c^
Fatigue	57 (48%)	61 (45%)	61 (45%)	0.8^c^
Diarrhea	21 (18%)	16 (12%)	25 (18%)	0.3^c^
Olfactory disturbance	29 (25%)	23 (17%)	37 (27%)	0.1^c^
Comorbidity				
Respiratory	11 (9%)	14 (10%)	13 (10%)	1.0^c^
Cardiovascular	46 (39%)^f^	32 (23%)^f^	20 (15%)^f^	< 0.001^c^
Malignancy	6 (51%)	8 (6%)	6 (4%)	1.0^c^
Diabetes	17 (14%)	9 (7%)	12 (9%)	0.1^c^
Smoking				
Never smoker	62 (53%)	79 (58%)	78 (57%)	0.8^c^
Current smoker	22 (19%)	27 (20%)	24 (18%)	–
Ex-smoker	34 (29%)	31 (23%)	34 (25%)	–

Data are shown as absolute numbers (percentage), mean ± standard deviation, median (interquartile range)

*Pt* patients, *DOB* dyspnea on breath

^a^One-way analysis of variance

^b^Kruskal–Wallis test

^c^Pearson *χ*^2^ test or Fisher’s exact test

^d^Bonferroni corrected *p* = 0.012 for wild type vs. Alpha variant, Bonferroni corrected *p* < 0.001 for wild type vs. Delta variant, and Bonferroni corrected *p* = 0.281 for Alpha variant vs. Delta variant

^e^Bonferroni corrected *p* = 1.0 for wild type vs. Alpha variant, Bonferroni corrected *p* = 0.003 for wild type vs. Delta variant, and Bonferroni corrected *p* = 0.033 for Alpha variant vs. Delta variant

^f^Bonferroni corrected *p* = 0.03 for wild type vs. Alpha variant, Bonferroni corrected *p* < 0.001 for wild type vs. Delta variant, and Bonferroni corrected *p* = 0.27 for Alpha variant vs. Delta variant

### CT findings

**Fig. 1:**
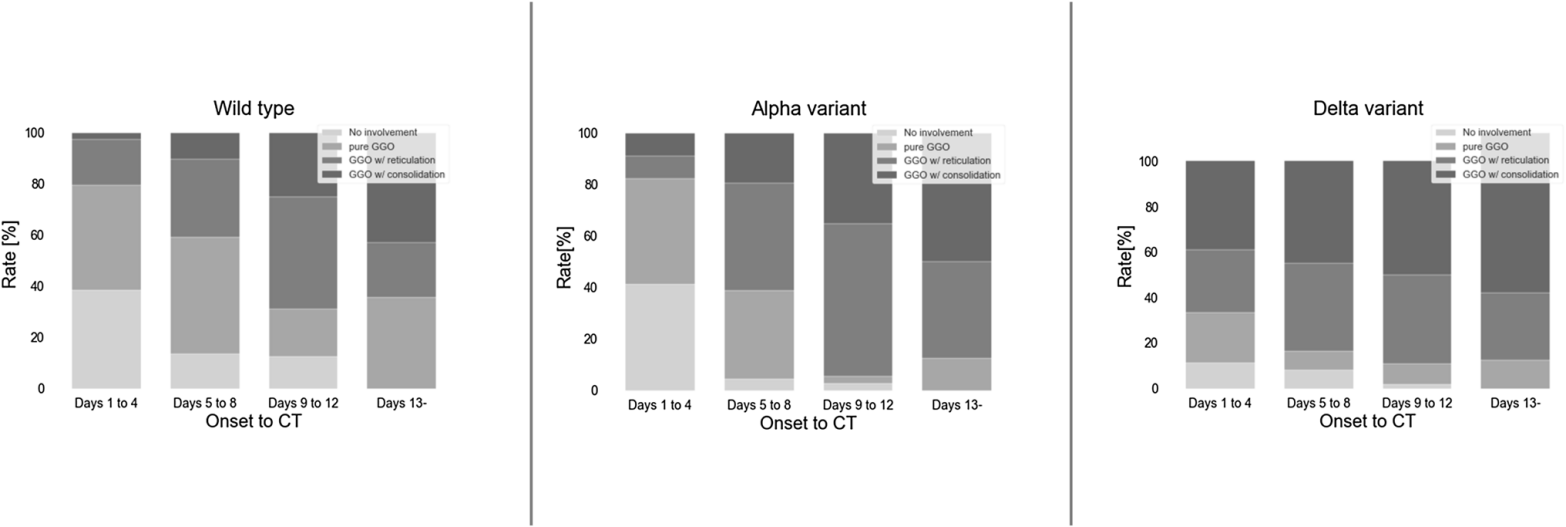
The percentage of chest CT primary change of each subgroup

The percentage of chest CT primary change was summarized in Fig. 1. Details of the CT findings were summarized in Tables 2, 3, 4, 5. After being stratified by chronological disease stages, patient age was still significantly different between the subgroups on days 1–4 (all, *p* = 0.03; wild type vs. Alpha variant, Bonferroni corrected *p* = 0.06; wild type vs. Delta variant, Bonferroni corrected *p* = 0.1, Alpha variant vs. Delta variant, Bonferroni corrected *p* = 1.0) on days 5–8 (all, *p* = 0.005; wild type vs. Alpha variant, Bonferroni corrected *p* = 0.4; wild type vs. Delta variant, Bonferroni corrected *p* = 0.003, Alpha variant vs. Delta variant, Bonferroni corrected *p* = 0.2). There was no statistically significant difference in clinical severity between the subgroups in any of the disease stages.

**Table 2:** Comparison of CT findings of each subtype on days 1–4

	Wild type (*n* = 39)	Alpha variant (*n* = 34)	Delta variant (*n* = 18)	*p *value
Age	60.1 ± 24.2^c^	46.7 ± 18.3^c^	45.5 ± 15.1^c^	0.03^a^
Clinical severity				
Mild	31 (82%)	30 (88%)	13 (73%)	0.3^b^
Moderate	8 (18%)	4 (12%)	5 (27%)	–
Primary changes				
Absent	15 (39%)^d^	14 (41%)^d^	2 (11%)^d^	0.002^b^
Pure GGO	16 (41%)	14 (41%)	4 (22%)	–
GGO w/reticulation	7 (18%)	3 (9%)	5 (28%)	–
GGO w/consolidation	1 (3%)	3 (9%)	7 (39%)	–
Repairing changes				
Absent	28 (72%)^e^	29 (75%)^e^	7 (39%)^e^	0.03^b^
Present	11 (28%)	5 (15%)	11 (61%)	–
Subpleural line	4 (10%)^f^	1 (3%)^f^	10 (56%)^f^	< 0.001^b^
Bronchus distortion	5 (13%)	2 (6%)	3 (17%)	0.4^b^
Fibrotic stripe	11 (28%)	5 (15%)	6 (33%)	0.2^b^
Subsidiary findings				
Reversed halo sign	0 (0%)	0 (0%)	0 (0%)	1.0^b^
Centrilobular nodule	4 (10%)	1 (3%)	0 (0%)	0.3^b^
Tree-in-bud	0 (0%)	1 (3%)	0 (0%)	0.5^b^
Pleural effusion	0 (0%)	1 (3%)	1 (6%)	0.3^b^
Lymphadenopathy	2 (5%)	1 (3%)	1 (6%)	1.0^b^
Fatty liver	4 (10%)	8 (24%)	4 (22%)	0.2^b^
Axial distribution				
Central	1 (3%)	1 (3%)	1 (6%)	0.7^b^
Peripheral	14 (36%)	16 (47%)	11 (61%)	–
Diffuse	9 (23%)	4 (12%)	4 (22%)	–
Involved lobes				
One lobe	5 (13%)	10 (29%)	2 (11%)	0.054^b^
Unilateral multilobe	3 (8%)	1 (3%)	0 (0%)	–
Bilateral multilobe	16 (41%)	10 (29%)	14 (78%)	–

Data are shown as absolute numbers (percentage), mean ± standard deviation

*GGO* Ground-glass opacity, *w* with

^a^One-way analysis of variance

^b^Pearson *χ*^2^ test or Fisher’s exact test

^c^Bonferroni corrected *p* = 0.06 for wild type vs. Alpha variant, Bonferroni corrected *p* = 0.1 for wild type vs. Delta variant, and Bonferroni corrected *p* = 1.0 for Alpha variant vs. Delta variant

^d^Bonferroni corrected *p* = 1.0 for wild type vs. Alpha variant, Bonferroni corrected *p* = 0.012 for wild type vs. Delta variant, and Bonferroni corrected *p* = 0.003 for Alpha variant vs. Delta variant

^e^Bonferroni corrected *p* = 0.8 for wild type vs. Alpha variant, Bonferroni corrected *p* = 0.3 for wild type vs. Delta variant, and Bonferroni corrected *p* = 0.033 for Alpha variant vs. Delta variant

^f^Bonferroni corrected *p* = 1.0 for wild type vs. Alpha variant, Bonferroni corrected *p* = 0.003 for wild type vs. Delta variant, and Bonferroni corrected *p* < 0.001 for Alpha variant vs. Delta variant

**Table 3:** Comparison of CT findings of each subtype on days 5–8

	Wild type (*n* = 59)	Alpha variant (*n* = 67)	Delta variant (*n* = 49)	*p* value
Age	54.8 ± 18.8^c^	50.5 ± 16.8^c^	43.8 ± 14.2^c^	0.005^a^
Clinical severity				
Mild	46 (78%)	45 (67%)	30 (61%)	0.2^b^
Moderate	13 (22%)	22 (33%)	19 (39%)	–
Primary changes				
Absent	8 (14%)^d^	3 (5%)^d^	4 (8%)^d^	< 0.001^b^
Pure GGO	27 (46%)	23 (34%)	4 (8%)	–
GGO w/reticulation	18 (31%)	28 (42%)	19 (39%)	–
GGO w/consolidation	6 (10%)	13(19%)	22 (45%)	–
Repairing changes				
Absent	35 (59%)^e^	35 (52%)^e^	10 (20%)^e^	< 0.001^b^
Present	24 (41%)	32 (48%)	39 (80%)	–
Subpleural line	18 (31%)	14 (21%)	38 (78%)	0.2^b^
Bronchus distortion	9 (15%)	23 (34%)	18 (37%)	0.4^b^
Fibrotic stripe	24 (41%)	32 (48%)	22 (45%)	0.2^b^
Subsidiary findings				
Reversed halo sign	0 (0%)	1 (2%)	2 (4%)	0.3^b^
Centrilobular nodule	1 (2%)	3 (5%)	1 (2%)	0.5^b^
Tree-in-bud	1 (2%)	0 (0%)	0 (0%)	1.0^b^
Pleural effusion	1 (2%)	3 (5%)	1 (2%)	0.7^b^
Lymphadenopathy	3 (5%)	1 (2%)	0 (0%)	0.3^b^
Fatty liver	7 (12%)	19 (28%)	10 (20%)	0.07^b^
Distribution				
Central	1 (2%)	1 (2%)	0 (0%)	0.2^b^
Peripheral	36 (61%)	37 (55%)	23 (47%)	–
Diffuse	15 (25%)	26 (39%)	22 (45%)	–
Involved lobes				
One lobe	3 (5%)	7 (10%)	3 (6%)	0.3^b^
Unilateral multilobe	4 (7%)	2 (3%)	0 (0%)	–
Bilateral multilobe	44 (75%)	55 (82%)	42 (86%)	–

Data are shown as absolute numbers (percentage), mean ± standard deviation

*GGO* Ground-glass opacity, *w* with

^a^One-way analysis of variance

^b^Pearson *χ*^2^ test or Fisher’s exact test

^c^Bonferroni corrected *p* = 0.4 for wild type vs. Alpha variant, Bonferroni corrected *p* = 0.003 for wild type vs. Delta variant, and Bonferroni corrected *p* = 0.2 for Alpha variant vs. Delta variant

^d^Bonferroni corrected *p* = 0.2 for wild type vs. Alpha variant, Bonferroni corrected *p* < 0.001 for wild type vs. Delta variant, and Bonferroni corrected *p* = 0.003 for Alpha variant vs. Delta variant

^e^Bonferroni corrected *p* = 1.0 for wild type vs. Alpha variant, Bonferroni corrected *p* < 0.001 for wild type vs. Delta variant, and Bonferroni corrected *p* < 0.001 for Alpha variant vs. Delta variant

**Table 4:** Comparison of CT findings of each subtype on days 9–12

	Wild type (*n* = 16)	Alpha variant (*n* = 37)	Delta variant (*n* = 56)	*p* value	
Age	56.2 ± 18.6	49.7 ± 14.6	45.4 ± 10	0.07^a^	
Clinical severity					
Mild	9 (56%)	20 (54%)	28 (50%)	0.9^b^	
Moderate	7 (44%)	17 (46%)	28 (50%)	–	
Primary changes					
Absent	2 (13%)	1 (3%)	1 (2%)	0.06^b^	
Pure GGO	3 (19%)	1 (3%)	5 (9%)	–	
GGO w/reticulation	7 (44%)	22 (60%)	22 (39%)	–	
GGO w/consolidation	4 (25%)	13 (35%)	28 (50%)	–	
Repairing changes					
Absent	6 (37%)^c^	10 (27%)^c^	2 (4%)^c^	0.001^b^	
Present	10 (63%)	27 (73%)	54 (96%)	–	
Subpleural line	4 (25%)^d^	16 (43%)^d^	50 (89%)^d^	< 0.001^b^	
Bronchus distortion	5 (31%)	20 (54%)	26 (46$)	0.4^b^	
Fibrotic stripe	10 (63%)	26 (70%)	31 (55%)	0.2^b^	
Subsidiary findings					
Reversed halo sign	1 (6%)	1 (3%)	2 (4%)	0.3^b^	
Centrilobular nodule	1 (6%)	0 (0%)	1 (2%)	0.5^b^	
Tree-in-bud	0 (0%)	0 (0%)	0 (0%)	1.0^b^	
Pleural effusion	0 (0%)	4 (11%)	3 (5%)	0.4^b^	
Lymphadenopathy	0 (0%)	0 (0%)	4 (7%)	0.2^b^	
Fatty liver	3 (19%)	13 (35%)	8 (14%)	0.06^b^	
Distribution					
Central	0 (0%)	1 (3%)	1 (2%)	0.3^b^	
Peripheral	8 (50%)	20 (54%)	21 (38%)	–	
Diffuse	6 (38%)	15 (41%)	33 (59%)	–	
Involved lobes					
One lobe	1 (6%)	0 (0%)	0 (0%)	0.1^b^	
Unilateral multilobe	0 (0%)	0 (0%)	0 (0%)	–	
Bilateral multilobe	13 (81%)	36 (97%)	55 (98%)	–	

Data are shown as absolute numbers (percentage), mean ± standard deviation

*GGO* Ground-glass opacity, *w* with

^a^One-way analysis of variance

^b^Pearson *χ*^2^ test or Fisher’s exact test

^c^Bonferroni corrected *p* = 1.0 for wild type vs. Alpha variant, *p* = 0.003 for wild type vs. Delta variant, and *p* = 0.003 for Alpha variant vs. Delta variant

^d^Bonferroni corrected *p* = 0.7 for wild type vs. Alpha variant, *p* < 0.001 for wild type vs. Delta variant, and *p* < 0.001 for Alpha variant vs. Delta variant

**Table 5:** Comparison of CT findings of each subtype on days 13 or more

	Wild type (*n* = 14)	Alpha variant (*n* = 8)	Delta variant (*n* = 17)	*p* value
Age	64.5 ± 20.0	68.9 ± 14.0	52.1 ± 13.0	0.7^a^
Clinical severity				
Mild	5 (36%)	0 (0%)	3 (18%)	0.1^b^
Moderate	9 (64%)	8 (100%)	14 (82%)	–
Primary changes				
Absent	0 (0%)	0 (0%)	0 (0%)	0.08^b^
Pure GGO	5 (36%)	1 (13%)	0 (0%)	–
GGO w/reticulation	3 (21%)	3 (38%)	5 (29%)	–
GGO w/consolidation	6 (43%)	4 (50%)	12 (71%)	–
Repairing changes				
Absent	2 (14%)	1 (14%)	1 (6%)	0.8^b^
Present	12 (86%)	7 (86%)	16 (94%)	–
Subpleural line	7 (50%)	3 (38%)	13 (77%)	0.1^b^
Bronchus distortion	8 (57%)	6 (75%)	12 (71%)	0.7^b^
Fibrotic stripe	11 (79%)	6 (75%)	14 (82%)	1.0^b^
Subsidiary findings				
Reversed halo sign	0 (0%)	0 (0%)	0 (0%)	1.0^b^
Centrilobular nodule	0 (0%)	0 (0%)	0 (0%)	1.0^b^
Tree-in-bud	0 (0%)	0 (0%)	0 (0%)	1.0^b^
Pleural effusion	2 (14%)	0 (0%)	4 (24%)	0.3^b^
Lymphadenopathy	2 (14%)	1 (13%)	3 (18%)	1.0^b^
Fatty liver	2 (14%)	2 (25%)	0 (0%)	0.09^b^
Distribution				
Central	0 (0%)	0 (0%)	0 (0%)	0.6^b^
Peripheral	5 (36%)	1 (13%)	4 (24%)	–
Diffuse	9 (64%)	7 (88%)	13 (77%)	–
Involved lobes				
One lobe	2 (14%)	0 (0%)	0 (0%)	0.2^b^
Unilateral multilobe	0 (0%)	0 (0%)	0 (0%)	–
Bilateral multilobe	11 (79%)	8 (100%)	17 (100%)	–

Data are shown as absolute numbers (percentage), mean ± standard deviation

*GGO* ground-glass opacity, *w* with

^a^One-way analysis of variance

^b^Pearson *χ*^2^ test or Fisher’s exact test

**Fig. 2:**
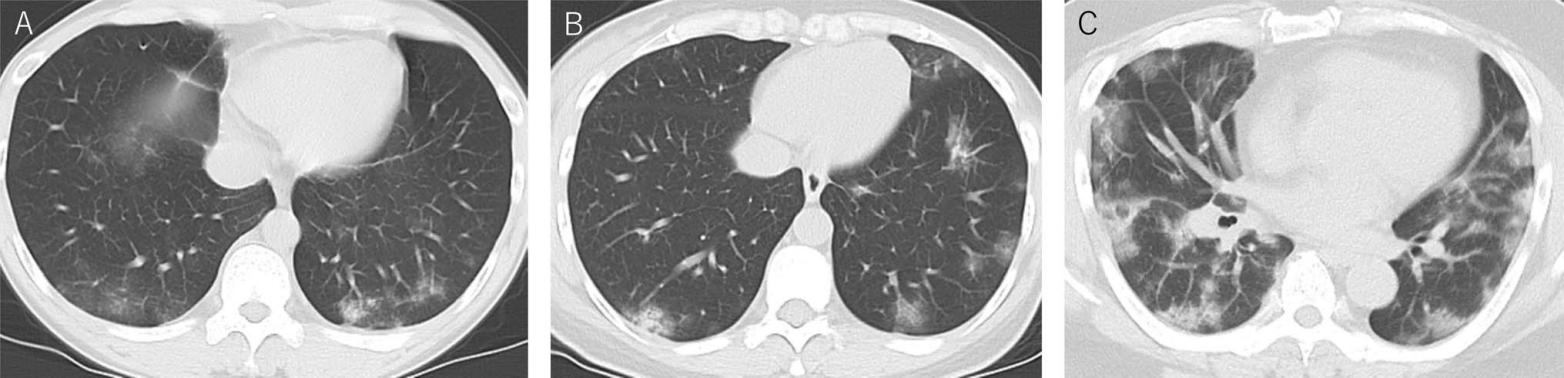
**A** A 46-year-old woman infected with SARS-CoV-2 wild type. On axial CT image on day 3, non-segmental ground-glass opacities were demonstrated in the left and right lower lobes. **B** A 39-year-old woman infected with SARS-CoV-2 Alpha variant. On axial CT image on day 3, non-segmental patchy ground-glass opacities were demonstrated in the left lower and upper lobes and right lower and middle lobes. **C** A 52-year-old man infected with SARS-CoV-2 Delta variant. On axial CT image on day 3, non-segmental ground-glass diffuse opacities were demonstrated in the left lower and upper lobes and right lower and middle lobes

Regarding primary changes, GGO with consolidation was more frequent in the order of Delta variant, Alpha variant, and wild type in all disease stages. The proportion of each primary change in each disease stage were as follows. On days 1–4, “no involvement” was observed in 39%, 41%, and 11%, respectively, in wild type, Alpha variant, and Delta variant; pure GGO in 41%, 41%, and 22%; GGO with reticulation in 18%, 9%, and 28%; and GGO with consolidation in 3%, 9%, and 39% (all, *p* = 0.002; wild type vs. Alpha variant, Bonferroni corrected *p* = 1.0; wild type vs. Delta variant, Bonferroni corrected *p* = 0.01; Alpha variant vs. Delta variant, Bonferroni corrected *p* = 0.003) (Table 2). On days 5–8, “no involvement” was observed in 14%, 5%, and 8%, respectively, in wild type, Alpha variant, and Delta variant; pure GGO in 46%, 34%, and 8%; GGO with reticulation in 31%, 42%, and 39%; and GGO with consolidation in 10%, 19%, and 45% (all, *p* < 0.001; wild type vs. Alpha variant, Bonferroni corrected *p* = 0.2; wild type vs. Delta variant, Bonferroni corrected *p* < 0.001; Alpha variant vs. Delta variant, Bonferroni corrected *p* = 0.003) (Table 3). On days 9–12, “no involvement” was observed in 13%, 3%, and 2%, respectively, in wild type, Alpha variant, and Delta variant; pure GGO in 19%, 3%, and 9%; GGO with reticulation in 44%, 60%, and 39%; and GGO with consolidation in 25%, 35%, and 50% (all, *p* = 0.06) (Table 4). On days 13 or more, “no involvement” was observed in 0%, 0%, and 0%, respectively, in wild type, Alpha variant, and Delta variant; pure GGO in 36%, 13%, and 0%; GGO with reticulation 21%, 38%, and 29%; and GGO with consolidation in 43%, 50%, and 71% (*p* = 0.08) (Table 5). Repairing changes were more frequent in the order of Delta variant, Alpha variant, and wild type in all disease stages. The proportion of repairing changes of the wild type, Alpha variant, and Delta variant were as follows: 28%, 15%, and 61% on days 1–4 (all, *p* = 0.03; wild type vs. Alpha variant, Bonferroni corrected *p* = 0.8; wild type vs. Delta variant, Bonferroni corrected *p* = 0.3; Alpha variant vs. Delta variant, Bonferroni corrected *p* = 0.03) (Table 2); 41%, 48%, and 80% on days 5–8 (all, *p* < 0.001; wild type vs. Alpha variant, Bonferroni corrected *p* = 1.0; wild type vs. Delta variant, Bonferroni corrected *p* < 0.001; Alpha variant vs. Delta variant, Bonferroni corrected *p* < 0.001) **(**Table 3); 63%, 73%, and 96% on days 9–12 (all, *p* = 0.001; wild type vs. Alpha variant, Bonferroni corrected *p* = 1.0; wild type vs. Delta variant, Bonferroni corrected *p* = 0.003; Alpha variant vs. Delta variant, Bonferroni corrected *p* = 0.003) (Table 4); and 86%, 86%, and 94% on days 13 or more (*p* = 0.815) (Table 5). There was no statistically significant difference in the presence of subsidiary findings including pleural effusion, lymphadenopathy, fatty liver, axial distribution of the opacities, and laterality of the involved lobes.

Chest CT severity scores of each subtype were summarized in Table 6. Median (with [interquartile range]) total chest CT scores of wild type, Alpha variant, and Delta variant were as follows: 2 [0–4], 1 [0–3.75], 6.5 [3–8] on days 1–4 (all, *p* = 0.008.; wild type vs. Alpha variant, Bonferroni corrected *p* = 1.0; wild type vs. Delta variant, Bonferroni corrected *p* = 0.01; Alpha variant vs. Delta variant, Bonferroni corrected *p* = 0.002); 5 [2.5–8], 7 [4–10], and 9 [5–11] on days 5–8 (all, *p* = 0.02; wild type vs. Alpha variant, Bonferroni corrected *p* = 0.2; wild type vs. Delta variant, Bonferroni corrected *p* = 0.005; Alpha variant vs. Delta variant, Bonferroni corrected *p* = 0.5); 7 [5.25–9.25], 8 [7–12], and 11 [10–14.25] on days 9–12 (*p* = 0.04); and 10 [8.7–14.5], 12 [10.6–14.75], and 13 [12.2–14] on days 13 or more (*p* = 0.7). Representative CT images of each subtype were shown in Fig. 2.

**Table 6:** Comparison of chest CT scores of each subtype

	Wild type (*n* = 128)	Alpha variant (*n* = 146)	Delta variant (*n* = 140)	*p* value
Days 1–4				
Total	2 [0–4]	1 [0–3.75]	6.5 [3–8]	0.002^a^
R total	1 [0–3]	0 [0–1.75]	4 [2–5]	< 0.001^b^
L total	1 [0–2]	0 [0–2]	2 [1.25–3.75]	0.007^c^
Days 5–8				
Total	5 [2.5–8]	7 [4–10]	9 [5–11]	0.007^d^
R total	3 [1–4.5]	4 [2–6]	5 [3–6]	0.009^e^
L total	2 [1–4]	3 [1–4]	4 [2–5]	0.016^f^
Days 9–12				
Total	7 [5.25–9.25]	8 [7–12]	11 [10–14.25]	0.02
R total	3.5 [3–4.25]	4 [4–7]	6 [5–8]	0.009^g^
L total	3 [2.25–5]	4 [3–5]	4.5 [4–6]	0.09
Days 13 or more				
Total	10 [8.7–14.5]	12 [10.6–14.75]	13 [12.2–14]	0.2
R total	5.5 [4.85–6.75]	7.5 [6.5–10]	6 [6–9]	0.07
L total	4.5 [3.85–6.75]	4 [4–5.5]	6 [5.2–6]	0.4

Data are shown as absolute median with (interquartile range), subgroup comparison was performed using Kruskal–Wallis test

*R* right, *L* left

^a^Bonferroni corrected *p* = 1.0 for wild type vs. Alpha variant, Bonferroni corrected *p* = 0.01 for wild type vs. Delta variant, and Bonferroni corrected *p* = 0.002 for Alpha variant vs. Delta variant

^b^Bonferroni corrected *p* = 0.3 for wild type vs. Alpha variant, Bonferroni corrected *p* = 0.02 for wild type vs. Delta variant, and Bonferroni corrected *p* < 0.001 for Alpha variant vs. Delta variant

^c^Bonferroni corrected *p* = 1.0 for wild type vs. Alpha variant, Bonferroni corrected *p* = 0.01 for wild type vs. Delta variant, and Bonferroni corrected *p* = 0.009 for Alpha variant vs. Delta variant

^d^Bonferroni corrected *p* = 0.2 for wild type vs. Alpha variant, Bonferroni corrected *p* = 0.005 for wild type vs. Delta variant, and Bonferroni corrected *p* = 0.4 for Alpha variant vs. Delta variant

^e^Bonferroni corrected *p* = 0.07 for wild type vs. Alpha variant, Bonferroni corrected *p* = 0.01 for wild type vs. Delta variant, and Bonferroni corrected *p* = 1.0 for Alpha variant vs. Delta variant

^f^Bonferroni corrected *p* = 0.7 for wild type vs. Alpha variant, Bonferroni corrected *p* = 0.2 for wild type vs. Delta variant, and Bonferroni corrected *p* = 0.01 for Alpha variant vs. Delta variant

^g^Bonferroni corrected *p* = 0.7 for wild type vs. Alpha variant, Bonferroni corrected *p* = 0.01 for wild type vs. Delta variant, and Bonferroni corrected *p* = 0.1 for Alpha variant vs. Delta variant

## Discussion

In this study, CT features and chest CT severity scores were compared between patients infected with wild type, Alpha variant, and Delta variant of SARS-CoV-2 by chronological disease stage. During the pandemic, the national healthcare policy has introduced changes in hospital admission criteria because of the upsurge in the number of COVID-19 cases, hospital occupancy rate, and increasing burden on the healthcare system. In the early period of the pandemic, confirmed patients were hospitalized for disease management and infection control irrespective of the disease severity; in the later period, such patients were instead recommended to stay home or at designated isolation facilities and not permitted to present to hospitals until their disease severity progressed to beyond the level requiring medical care. It is reasonable to assume that such changes may have influenced the background status of the admitted patients. As predicted, significant differences were observed in age, clinical severity, and cardiovascular comorbidity between the subgroups. To minimize the selection bias due to the different inclusion periods, the CT findings were compared after stratification by chronological disease stage.

Within subgroup analysis revealed the temporal changes of the CT pattern to be roughly similar in the three subtypes. Along the disease course, the proportion of “no involvement” or pure GGO, which are known as early CT findings of COVID-19, decreased while GGO with reticulation or consolidation, which are known as later CT findings, increased in all the subtypes. Repairing changes, which was also known as CT findings of the absorptive stage, also increased along the disease course in all  the subtypes. These CT patterns characterize the typical evolution of wild type COVID-19, in which the number of lesions and extent and density of the opacities increase with disease progression until peaking around about days 10–14 when absorptive findings become predominant [13–20]. Extent of involvement also increased from relatively focal in the early stage to more extensive and bilateral opacities later along with total CT severity scores, in consistent with the findings of previous reports [7] Total CT severity scores also increased along the disease course within the time span evaluated in this study. One salient finding of this study was that temporal CT patterns of wild type COVID-19 were also applicable to those of Alpha and Delta variants.

Notably, the CT findings that commonly appear in the later stages of COVID-19 (i.e., GGO w/ reticulation or consolidation) were already more dominant than the early CT findings (i.e., “no involvement” or pure GGO) along with conspicuous repairing changes in as early as days 1–4 in Delta variant. These findings contrasted with those of wild type or Alpha variant, in which “no involvement” or pure GGO was predominant in the early stages. Given that there was no significant difference in their clinical severity, it may be hypothesized that the pneumonia progression of Delta variant may be faster than that of the other two. Between subgroup analysis may reinforce this hypothesis with the results that the proportion of GGO with consolidation was significantly more frequent in Delta variant in the early two stages. Repairing changes were also frequent in Delta variant rising to close to statistical significance on days 1–4 and significantly frequent than the other two in the early two stages. Disease extent as evaluated by total chest CT severity score of Delta variant was also significantly greater than that of the other two on days 1–4 and still significantly greater than that of wild type on days 5–8. A virological study showed that enhanced pathogenicity of Delta variant may be ascribed to mutations in the spike protein that enhance fusogenicity and facilitates expansion more efficiently in the human body through cell–cell fusion than the wild type and Alpha variant [21]. In contrast, there was no significant radiological difference between wild type and Alpha variant. This was also consistent with a previous report from the UK that compared the chest CT findings of these subtypes [8].

This study is subject to limitations and challenges. First, because of its retrospective nature, a selection bias may have been introduced. Therefore, CT findings were compared after being stratified by chronological disease stages, with confirmed clinical severities then found not to differ significantly between the subgroups. Second, although virus genome data of the individual patients was not available, they were deemed to be infected with different subtypes of COVID-19 by separating the inclusion period according to the government’s official surveillance report. However, only weekly total numbers were available in the surveillance report. Therefore, some overlaps were present in the transitional periods of different variants. For example, the proportion of each subtype was 51.2%, 33.7%, and 15.1% for Alpha variant, Delta variant, and wild type, respectively, in the week of July 12–18, 2021 and 33.3%, 53.1%, and 13.5%, respectively, in the week of July 19–25, 2021. However, their impacts were minimal because the proportion of the number of cases included in these transitional periods were small. Therefore, it is reasonable to consider that the purpose of this article to reveal a rough tendency among different subtypes was achieved despite of this limitation. Third, the evaluation of this study was limited to patients with mild or moderate disease severity because of insufficient sample size and may not be applicable to those with severe disease requiring ICU admission or mechanical ventilation. Fourth, the impact of this study may also have suffered from having been conducted at a single institution and limited to a single ethnic group. Further study will be needed to validate the results of this study with more diverse populations.

## Conclusions

In conclusion, COVID-19 pneumonia caused by Delta variant showed GGO with reticulation or consolidation and repairing changes more conspicuously than did wild type and Alpha variant in the early phase. Chest CT scores of Delta variant were also higher than those of wild type during days 1–8 and Alpha variant during days 1–4. This suggests that the progression of COVID-19 pneumonia of Delta variant may be more rapid than in the other two. In contrast, there was no evidence of any difference in the CT findings between wild type and Alpha variant. The results of this study may be valuable for the ongoing and future battle with COVID-19 variants, reminding that different CT patterns may predispose different subtype infection and different clinical course. That is, a rapid progression on CT may be associated with the pathogenicity of viral subtype, which in turn could be applicable to newly emerging viral strains and useful for predicting the pathogenicity of the virus and defining the frequency of CT monitoring or criteria of hospitalization, ICU admission, or early intervention before accumulation of evidence regarding the new virus subtype.
